# A multicentre comparative study of serological methods for diagnosing Chagas disease in Brazil

**DOI:** 10.1590/0074-02760240282

**Published:** 2025-06-13

**Authors:** Alejandro Luquetti Ostermayer, Fernanda Alvarenga Cardoso Medeiros, Jacqueline Araújo Domingos Iturra, Job Alves de Souza Filho, Leonardo Maia Leony, Larissa de Carvalho Medrado Vasconcelos, Liliane da Rocha Siriano, Suelene Brito do Nascimento Tavares, Vinícius Silva Belo, Andréa Silvestre de Sousa, Fred Luciano Neves Santos

**Affiliations:** 1 Universidade Federal de Goiás, Hospital Universitário, Núcleo de Estudos em Doença de Chagas, Goiânia, GO, Brasil; 2 Fundação Ezequiel Dias, Serviço de Doenças Parasitárias, Belo Horizonte, MG, Brasil; 3 Fundação Oswaldo Cruz-Fiocruz, Instituto Gonçalo Moniz, Laboratório Avançado de Saúde Pública, Salvador, BA, Brasil; 4 Fundação Oswaldo Cruz-Fiocruz, Instituto Gonçalo Moniz, Grupo de Pesquisa Interdisciplinar em Biotecnologia e Epidemiologia de Doenças Infecciosas, Salvador, BA, Brasil; 5 Universidade Federal de São João Del-Rei, Divinópolis, MG, Brasil; 6 Fundação Oswaldo Cruz-Fiocruz, Instituto Nacional de Infectologia Evandro Chagas, Rio de Janeiro, RJ, Brasil; 7 Fundação Oswaldo Cruz-Fiocruz, Vice-Presidência de Pesquisa e Coleções Biológicas, Programa de Pesquisa Translacional em Doenças de Chagas, Rio de Janeiro, RJ, Brasil

**Keywords:** Chagas disease, immunodiagnosis, serological tests, sensitivity, specificity

## Abstract

**BACKGROUND:**

Chagas disease (CD), a neglected tropical disease caused by *Trypanosoma cruzi*, remains a significant often underdiagnosed public health challenge in endemic regions, affecting millions globally. Accurate and timely diagnosis is critical, but the performance of existing diagnostic methods varies widely in sensitivity and specificity.

**OBJECTIVES:**

This multicentre study assessed the diagnostic performance of 17 serological assays for detecting anti-*T. cruzi* antibodies.

**METHODS:**

Commercial enzyme immunoassays (EIA), indirect haemagglutination assays (IHA), indirect immunofluorescence assays (IIF), rapid diagnostic tests (RDT), and a chemiluminescent microparticle immunoassay (CMIA) were included in this study.

**FINDINGS:**

Some EIA-based tests achieved 100% sensitivity, while IHAs and IIFs demonstrated reduced specificity. CMIA exhibited 100% sensitivity, highlighting its potential as a robust screening tool. Combining EIAs with IHAs or IIFs improved overall sensitivity, often surpassing 99%, although specificity remained variable. Cross-reactivity with other parasitic diseases posed challenges to specificity, particularly in assays employing crude antigens.

**MAIN CONCLUSIONS:**

These findings emphasise the importance of tailoring diagnostic tool selection to regional epidemiological contexts and advancing antigen refinement to enhance diagnostic accuracy and accessibility, particularly in resource-limited settings.

Chagas disease (CD), also known as American trypanosomiasis, is a systemic parasitic infection caused by the flagellate protozoan *Trypanosoma cruzi*. Recognised as a neglected tropical disease, CD is endemic to 21 countries in the Americas but has been reported in 41 countries worldwide, representing a significant public health challenge. The disease causes an estimated 12,000 deaths annually, with 6 to 7 million individuals worldwide affected by the parasite.^1^ Despite its substantial mortality and morbidity, only 7% of *T. cruzi*-infected individuals are diagnosed, and fewer than 1% receive etiological treatment.^2^

The diagnostic process for CD is complex, requiring clinical evaluation, epidemiological data, and laboratory testing. During the acute phase, parasitological and molecular biology-based methods are employed, whereas the chronic phase relies on serological techniques, including indirect immunofluorescence (IFF), indirect haemagglutination (IHA), enzyme immunoassay (EIA), chemiluminescence microparticle immunoassays (CMIA), and rapid diagnostic tests (RDT).^3^ To maximise sensitivity and minimise undiagnosed cases, the diagnostic standard involves performing two serological tests in parallel, using distinct methodologies and antigens.^3^ ^,^ ^4^ While these tests generally achieve high sensitivity and specificity, variations in their accuracy and execution can lead to discrepancies. In cases of discordant results, retesting is advised, with a third test used to confirm the diagnosis if inconsistencies persist.^3^

Chagas disease disproportionately affects vulnerable populations in endemic regions, where limited access to diagnostic tools delays timely detection and treatment. This exacerbates the disease burden and perpetuates socioeconomic inequalities. Recognising the variability in diagnostic accuracy among existing methods and the urgent need for reliable diagnostics, our group previously assessed the performance of all RDTs licensed and commercially available in Brazil.^5^ However, a comparable analysis of other diagnostic platforms, such as EIA, IIF, IHA, and CMIA, remains lacking.

To address this gap, we conducted a comprehensive evaluation of all EIA, IIF, and IHA kits licensed for use in Brazil, alongside the chemiluminescent assay Architect Chagas, using the same sample panel from our prior study.^5^ This study aims to provide a thorough assessment of these diagnostic tools, contributing to improved diagnostic accuracy and supporting efforts to combat this neglected tropical disease.

## MATERIALS AND METHODS

*Ethical statements* - This multicentric study was approved by the Institutional Review Boards (IRBs) for Human Research of the following institutions: the Gonçalo Moniz Institute (IGM-FIOCRUZ), Salvador, Bahia, Brazil (CAAE 67809417.0.0000.0040), the Ezequiel Dias Foundation (FUNED), Belo Horizonte, Minas Gerais, Brazil (CAAE 21538619.4.2002.9507), and the Federal University of Goiás (UFG), Goiânia, Goiás, Brazil (CAAE 21538619.4.2001.5078). All patient data were anonymised to maintain confidentiality, and the requirement for written informed consent was waived by the ethics committees.

*Selection of diagnostic kits* - This study included all serological tests commercially available and registered with the Brazilian Health Regulatory Agency (ANVISA) in 2019, the time of project’s execution (Table I). A total of 17 kits met these inclusion criteria, comprising seven EIA kits: Biolisa Chagas Recombinante^®^ (Quibasa Química Básica Ltda, Belo Horizonte, Brazil), *T. cruzi* Ab - ELISA^®^ (Diapro Diagnostic Bioprobes SRL, Milan, Italy), Chagatest ELISA recombinante v. 4.0^®^ (Wiener Lab SAIC, Rosário, Argentina), Chagatest ELISA lisado^®^ (Wiener Lab SAIC, Rosário, Argentina), Anti Chagas SYM^®^ (Symbiosis Diagnostica Ltda, Leme, Brazil), Anti *Trypanosoma cruzi* IgG^®^ (Euroimmun Medizinische Labordiagnostika AG, Lübeck, Germany), and Gold ELISA Chagas^®^ (REM Indústria e Comércio Ltda, São Paulo, Brazil).

**TABLE I:** Commercial serological tests for detecting anti-*Trypanosoma cruzi* antibodies used in this study

Acronym	Name	Manufacturer	Diagnostic platform	ANVISA^*^ registry
EIABio	Biolisa Chagas Recombinante	Quibasa Química Básica (Brazil)	EIA	10269360306
EIADia	*T. cruzi* Ab - ELISA	Diapro Diagnostic Bioprobes SRL (Italy)	EIA	80345000187
EIARec	Chagatest ELISA recombinante v. 4.0	Wiener Lab (Argentina)	EIA	10162410454
EIALis	Chagatest ELISA lisado	Wiener Lab (Argentina)	EIA	10268590308
EIASym	Anti Chagas SYM	Symbiosis Diagnostica (Brazil)	EIA	80105220094
EIAEur	Anti *Trypanosoma* cruzi IgG	Euroimmun Medizinische Labordiagnostika (Germany)	EIA	81148560013
EIAGol	Gold ELISA Chagas	REM Indústria e Comércio (Brazil)	EIA	10162410454
IHAWie	Chagatest HAI screening A-V	Wiener Laboratórios (Argentina)	IHA	10268590223
IHAGol	Chagas-HAI	Gold Analisa Diagnóstica (Brazil)	IHA	80022230163
IIFVir	Chagas IFA IgG+IgM	Vircell SL (Spain)	IIF	80263710031
IIFCon	Imuno-Con Chagas	Wama Diagnóstica (Brazil)	IIF	10310030077
IIFBio	IIF Chagas	Bio-Manguinhos (Brazil)	IIF	80142170020
RDTOns	OnSite Chagas Ab Combo Rapid Test	CTK Biotech (USA)	RDT	80524900061
RDTSdb	SD Bioline Chagas AB	Abbott Diagnostics (USA)	RDT	10071770690
RDTWlc	WL Check Chagas	Wiener Lab (Argentina)	RDT	10268590323
RDTBio	TR Chagas Bio-Manguinhos	Bio-Manguinhos (Brazil)	RDT	80142170043
CMIAAr	Architect Chagas	Abbott Diagnostics (Germany)	CMIA	80146501745

^*^ ANVISA (Portuguese acronym for the Brazilian Health Regulatory Agency).

Additionally, two IHA kits were included: Chagatest HAI screening A-V^®^ (Wiener Laboratórios SAIC, Rosário, Argentina) and Chagas-HAI^®^ (Gold Analisa Diagnóstica LTDA, Brazil). The study also evaluated three IIF kits: Chagas IFA IgG+IgM^®^ (Vircell SL, Granada, Spain), Imuno-Con Chagas^®^ (Wama Diagnóstica, São Carlos, Brazil), and IIF Chagas^®^ (Bio-Manguinhos, Fiocruz, Rio de Janeiro, Brazil). Furthermore, four RDTs were part of the study: OnSite Chagas Ab Combo Rapid Test^®^ (CTK Biotech, Poway, United States), SD Bioline Chagas AB^®^ (Abbott, Chicago, United States), WL Check Chagas^®^ (Wiener Lab SAIC, Rosário, Argentina), and TR Chagas Bio-Manguinhos^®^ (Bio-Manguinhos, Fiocruz, Rio de Janeiro, Brazil). Detailed descriptions and diagnostic performance assessments of these RDTs are available in a previous study.^5^

The study also included the chemiluminescent microparticle immunoassay Architect Chagas^®^ (Abbott Diagnostics, Wiesbaden, Germany). All diagnostic kits were provided by the Oswaldo Cruz Foundation and distributed to participating reference laboratories by the General Coordination of Public Health Laboratories (CGLAB, Ministry of Health, Brazil) via a commercial shipping service. To ensure consistency and reliability, all kits from each manufacturer were sourced from the same batch.

*Participating reference laboratories* - The study was conducted at three Brazilian reference laboratories: the Advanced Public Health Laboratory (LASP) at the Gonçalo Moniz Institute (FIOCRUZ) in Salvador, Bahia; the Parasitic Diseases Service of the FUNED in Belo Horizonte, Minas Gerais; and the Chagas Disease Study Centre (NEDoC) at the UFG in Goiânia, Goiás. EIA, IHA, and IIF kits were evaluated at FUNED and NEDoC, while RDTs were analysed at all three laboratories. The CMIA evaluation was conducted exclusively at FUNED. All laboratories adhered to Good Laboratory Practices. The same serum samples were analysed by all laboratories, with reactivity reconfirmed through conventional serology after thawing at the originating laboratory.

*Sample collection* - The sample size was determined using a 2% absolute error, with expected sensitivity and specificity of 99% and 99.5%, respectively, at a 95% confidence level. Calculations with OpenEpi^6^ indicated the need for a minimum of 96 anti-*T. cruzi*-positive and 58 anti-*T. cruzi*-negative serum samples. A total of 170 samples were analysed: 111 anti-*T. cruzi*-positive and 59 anti-*T. cruzi*-negative sera obtained from the NEDoC sera bank, diagnosed based on the clinical and laboratory criteria described below. To evaluate cross-reactivity, an additional 20 sera were included from individuals diagnosed with other infectious diseases based on parasitological or serological criteria. These sera, sourced from the FUNED sera bank, comprised samples from individuals with toxoplasmosis (n = 4), mucocutaneous leishmaniasis (n = 6), and visceral leishmaniasis (n = 10). As previously described,^5^ the anti-*T. cruzi*-positive samples were collected from individuals in the chronic phase of CD with documented epidemiological and clinical data, typically involving cardiac disease, megacolon, and/or megaoesophagus. Physicians in Goiás State referred these infected and uninfected individuals to the laboratory for diagnosis confirmation. This sample set primarily included sera with low or moderate reactivity in serological tests, such as titters below 1:640 in IIF and IHA, and reactivity indices between 1.2 and 2.0 (low reactivity) or 2.1 to 3.0 (moderate reactivity) in conventional EIA.

The selection of the 111 serum samples from infected individuals was primarily based on low to medium antibody concentrations. All individuals had appropriate clinical classification based on electrocardiograms and contrast X-ray evaluations of the oesophagus and colon. Among them, 36 (32.4%) were in the asymptomatic form; 29 (26.1%) had megaoesophagus and/or megacolon; 19 (17.1%) presented with cardiopathy (mainly right bundle branch block); 18 (16.2%) exhibited non-specific ECG abnormalities, and 9 (8.1%) had the mixed (cardiodigestive) form. The infected individuals included in this study originated from multiple Brazilian states. Of the total, 40 (36%) were born and residing in Goiás State, while 33% were from Bahia. Additional participants were from Minas Gerais (n = 9) and Tocantins (n = 2). Furthermore, 21 individuals (18.9%) were distributed across Pará, Maranhão, Piauí, Ceará, Paraíba, Pernambuco, and Rio Grande do Norte, with 1 to 5 individuals from each state.

All samples were thawed at -20ºC without additional preservatives and had been previously tested for *T. cruzi* infection using multiple diagnostic methods, including indirect immunofluorescence (anti-human IgG conjugated to fluorescein, Biomerieux Marcy L’Etoile), indirect haemagglutination (Chagatest HAI screening A-V^®^, Wiener lab, Rosario, Argentina), EIA Chagas III (BIOSChile, Ingeniería Genética SA, Santiago, Chile), and chemiluminescence microparticle immunoassay (Architect Chagas, Abbott Laboratories, Abbott GmbH, Wiesbaden, Germany). To maintain blinding, the samples were aliquoted and coded before being sent to the participating laboratories. The aliquots were stored on dry ice and transported by the CGLAB via a commercial shipping service. Supplementary data (Table) provides the serological results for each sample using the various diagnostic techniques.

*Serological assay execution* - All serological assays were conducted in accordance with the manufacturers’ technical guidelines. Each participating reference laboratory evaluated the same set of sera. Results were independently reviewed by two observers per institution. In cases of disagreement or uncertainty, a third observer was consulted, and if consensus could not be reached, the tests were repeated to ensure accuracy.

Test results were submitted exclusively to the sera bank supervisor, who had access to the serological profiles of the samples. Rapid diagnostic tests lacking control lines were classified as invalid. Final results were compiled through consensus among the laboratories and compared with the established serological profiles to evaluate the performance of each diagnostic kit.

*Usability assessment* - The usability of the serological tests was evaluated by assessing the ease of test performance and result interpretation. At the conclusion of the study, the technical staff responsible for conducting the tests completed a usability questionnaire for each kit. The questionnaire was adapted from previously published standardised questionnaires and included subjective assessments of qualitative variables.^7^ ^,^ ^8^ ^,^ ^9^

The variables assessed included the number of invalid tests, shelf life, storage temperature, volume of blood/serum/plasma required, number of procedural steps, time needed to perform the test, result stability, additional materials required, ease of package opening, ease of test execution, clarity in identifying reagents, quality of the instructions for use, and cost.

Each item in the questionnaire was scored individually, with higher scores indicating more favourable usability. A total of nine items were evaluated, with a maximum possible score of 27 points per kit.

*Statistical analysis* - Statistical methods were used to evaluate the performance of each diagnostic kit, determining parameters such as sensitivity, specificity, accuracy, likelihood ratios, and diagnostic odds ratios using a 2×2 contingency table. Confidence intervals (CIs) were calculated at a 95% confidence level (95% CI), with non-overlapping CI indicating statistical significance.

To comprehensively assess diagnostic performance, combined and sequential (serial) testing strategies were employed. In combined testing, two tests were performed simultaneously, and both had to be positive to indicate disease presence. Sequential testing involved a stepwise approach, where subsequent tests were administered based on prior results, with all tests requiring positive outcomes to confirm the diagnosis.^10^

Agreement between the results of all kits and the serological profiles of the samples was measured using Cohen’s Kappa coefficient (κ), categorised as follows: poor (κ = 0), slight (0 < κ ≤ 0.20), fair (0.21 < κ ≤ 0.40), moderate (0.41 < κ ≤ 0.60), substantial (0.61 < κ ≤ 0.80), and almost perfect (0.81 < κ ≤ 1.0).^11^ Performance parameters were analysed using MedCalc for Windows v. 20.190 (MedCalc Software, Ostend, Belgium), and graphical representations were created with GraphPad Prism 9.5.1 (GraphPad Software, San Diego, CA, USA).

To address diagnostic uncertainty and optimise sensitivity and specificity, pairwise analyses of individual test data were conducted following the recommendations of the World Health Organization (WHO) and the Pan American Health Organization (PAHO).^4^ Three scenarios involving the simultaneous use of two different diagnostic platforms were explored.

A graphical analysis assessed each kit’s proximity to an ideal test with 100% accuracy, plotting sensitivity on the y-axis and specificity on the x-axis. Four quadrants were proposed: Quadrant I (high sensitivity and specificity), Quadrant II (high sensitivity, low specificity), Quadrant III (low sensitivity and specificity), and Quadrant IV (low sensitivity, high specificity). PAHO guidelines for DC diagnostics specify the following minimum sensitivity and specificity standards: EIA kits (97% sensitivity, 98% specificity), RDTs (94% sensitivity, 97% specificity), and CMIA (99% sensitivity, 98% specificity).^4^ As no minimum values were provided for IHA and IIF, these were analysed alongside EIA kits.

## RESULTS

*Performance analysis* - Table II summarises the performance metrics for all 17 diagnostic kits analysed. Among the EIA kits, EIABio and EIASym achieved the highest sensitivity (100%), while EIAEur showed the lowest sensitivity (92.9%). Notably, EIAEur’s sensitivity was significantly lower than that of EIABio and EIASym, as indicated by non-overlapping 95% CI. No significant differences in sensitivity were observed among the other EIA kits.

**TABLE II:** Performance parameters obtained for the 18 kits licensed for use and commercially available in Brazil, as used in this study

Kits	SEN % (IC95%)	SPE % (IC95%)	ACC % (IC95%)	PLR	NLR	DOR	*K* (IC95%)
EIABio	100 (96.6-100)	86.8 (77.4-92.7)	94.6 (90.3-97.0)	7.6	0.0001	71,226	0.89 (0.82-0.95)
EIADia	99.0 (94.8-99.8)	90.5 (81.7-95.3)	95.5 (91.5-97.7)	10.5	0.0105	994	0.91 (0.84-0.97)
EIARec	99.1 (95.1-99.8)	80.8 (70.7-88.0)	91.5 (86.7-94.7)	5.2	0.0112	462	0.82 (0.74-0.90)
EIALis	99.1 (95.0-99.8)	78.2 (67.8-85.9)	90.4 (85.4-93.9)	4.5	0.0116	391	0.80 (0.71-0.89)
EIASym	100 (96.6-100)	88.5 (79.5-93.8)	95.2 (91.1-97.5)	8.7	0.0001	84,260	0.90 (0.84-0.96)
EIAEur	92.9 (86.0-96.5)	89.5 (80.6-94.6)	91.4 (86.3-94.7)	8.8	0.0798	111	0.82 (0.74-0.91)
EIAGol	94.5 (88.5-97.5)	86.8 (77.4-92.7)	91.4 (86.4-94.6)	7.2	0.0634	113	0.82 (0.74-0.90)
IHAWie	93.7 (87.6-96.9)	70.9 (60.1-79.7)	84.2 (78.4-88.7)	3.2	0.0890	36	0.67 (0.56-0.78)
IHAGol	71.2 (62.1-78.8)	94.9 (87.7-98.0)	81.1 (74.9-86.0)	14.1	0.3037	46	0.63 (0.52-0.74)
IIFVir	100 (96.5-100)	62.1 (50.1-72.8)	85.5 (79.5-90.0)	2.6	0.0002	17,547	0.67 (0.55-0.79)
IIFCon	87.4 (79.9-92.3)	88.6 (79.7-93.9)	87.9 (82.5-91.8)	7.7	0.1423	54	0.75 (0.66-0.85)
IIFBio	90.1 (83.1-94.4)	73.4 (62.8-81.9)	83.2 (77.2-87.8)	3.4	0.1350	25	0.65 (0.54-0.76)
RDTOns	92.8 (86.3-96.8)	87.3 (78.0-93.8)	90.5 (85.5-93.9)	7.3	0.08	89	0.80 (0.72-0.78)
RDTSdb	95.5 (89.9-98.5)	89.9 (81.0-95.5)	93.2 (88.6-96.0)	9.43	0.05	188	0.86 (0.78-0.93)
RDTWlc	97.3 (92.3-99.4)	92.4 (84.2-97.2)	95.3 (91.2-97.5)	12.8	0.03	438	0.90 (0.84-0.96)
RDTBio	100 (96.7-100)	78.5 (68.2-86.1)	91.1 (86.1-94.3)	4.65	<0.001	40,469	0.81 (0.72-0.90)
CMIAAr	97.3 (92.4-99.1)	81.4 (69.6-89.3)	91.8 (86.7-95.0)	5.2	0.03	157	0.81 (0.72-0.91)

SEN: sensitivity; SPE: specificity; ACC: accuracy; PLR: positive likelihood ratio; NLR: negative likelihood ratio; DOR: diagnostic odds ratio; *k*: Cohen’s *Kappa* index. Note: to determine the performance parameters, the number of samples with indeterminate results was disregarded.

For IHA, IHAWie and IHAGol identified 104 and 79 *T. cruzi*-positive samples, corresponding to sensitivities of 93.7% and 71.2%, respectively. The difference in sensitivity between these two kits was statistically significant. Among the IIF, IIFVir exhibited 100% sensitivity, outperforming IIFBio (90.1%) and IIFCon (87.4%). While IIFVir had significantly higher sensitivity compared to the other IIF tests, no significant differences were found between IIFBio and IIFCon. Sensitivity values for RDTs were reported previously^5^ but are included in Table II for comparison. RDTBio achieved the highest sensitivity (100%), followed by RDTWlc (97.3%), RDTSdb (95.5%), and RDTOns (92.8%), with no statistically significant differences among them. Lastly, the CMIAAr identified 97.3% of *T. cruzi*-positive samples.

In terms of specificity, EIA kits demonstrated lower performance, ranging from 78.2% (EIALis) to 90.5% (EIADia). No significant differences in specificity were observed among the EIA kits (Table II). Similarly, IHAWie exhibited low specificity (70.9%), whereas IHAGol achieved a higher specificity (94.9%), showing a significant difference between the two. Among the IIF tests, specificity values were also low, ranging from 62.1% (IIFVir) to 88.6% (IIFCon). A significant difference was observed between IIFCon and IIFVir, but not between IIFCon and IIFBio or IIFBio and IIFVir. For RDTs and CMIAAr, specificity values were lower than sensitivity values, ranging from 78.5% (RDTBio) to 92.4% (RDTWlc), with no significant differences among them. CMIAAr achieved a specificity of 81.4%.

In general, most tests demonstrated accuracy values exceeding 90% (Table II). The highest accuracy values were observed for two EIA kits, EIADia (95.5%) and EIASym (95.2%), as well as the RDT RDTWlc (95.3%). When diagnostic platforms were analysed collectively, no significant differences in accuracy were detected. However, EIABio, EIADia, and EIASym showed significantly higher accuracy compared to any IHA, as well as IIFVir and IIFBio. No other significant differences in accuracy were observed among the remaining tests.

Positive and negative likelihood ratios are presented in Table II. The highest positive likelihood ratios were observed for IHAGol, RDTWlc, and EIADia, while negative likelihood ratios were below 0.1 for most tests, except for IIFCon and IIFBio. Diagnostic odds ratios (DORs) were calculated based on these likelihood ratios. High DOR values were observed for EIABio (DOR = 71,226), EIASym (DOR = 84,260), RDTBio (DOR = 40,469), and IIFVir (DOR = 17,547). Lower, yet notable, DOR values were found for EIADia (DOR = 994). Among the IHA kits, lower DOR values were reported for IHAWie (DOR = 36) and IHAGol (DOR = 46). Overall, all other kits exhibited DOR values above 500.

Cohen’s Kappa analysis indicated almost perfect agreement with reference tests for five EIA kits (EIABio, EIADia, EIARec, EIASym, and EIAGol), three RDTs (RDTSdb, RDTWlc, and RDTBio), and the chemiluminescent assay (CMIAAr). Substantial agreement was observed for EIALis, IHAWie, IHAGol, IIFVir, IIFCon, IIFBio, and RDTOns.

**Fig. 1:**
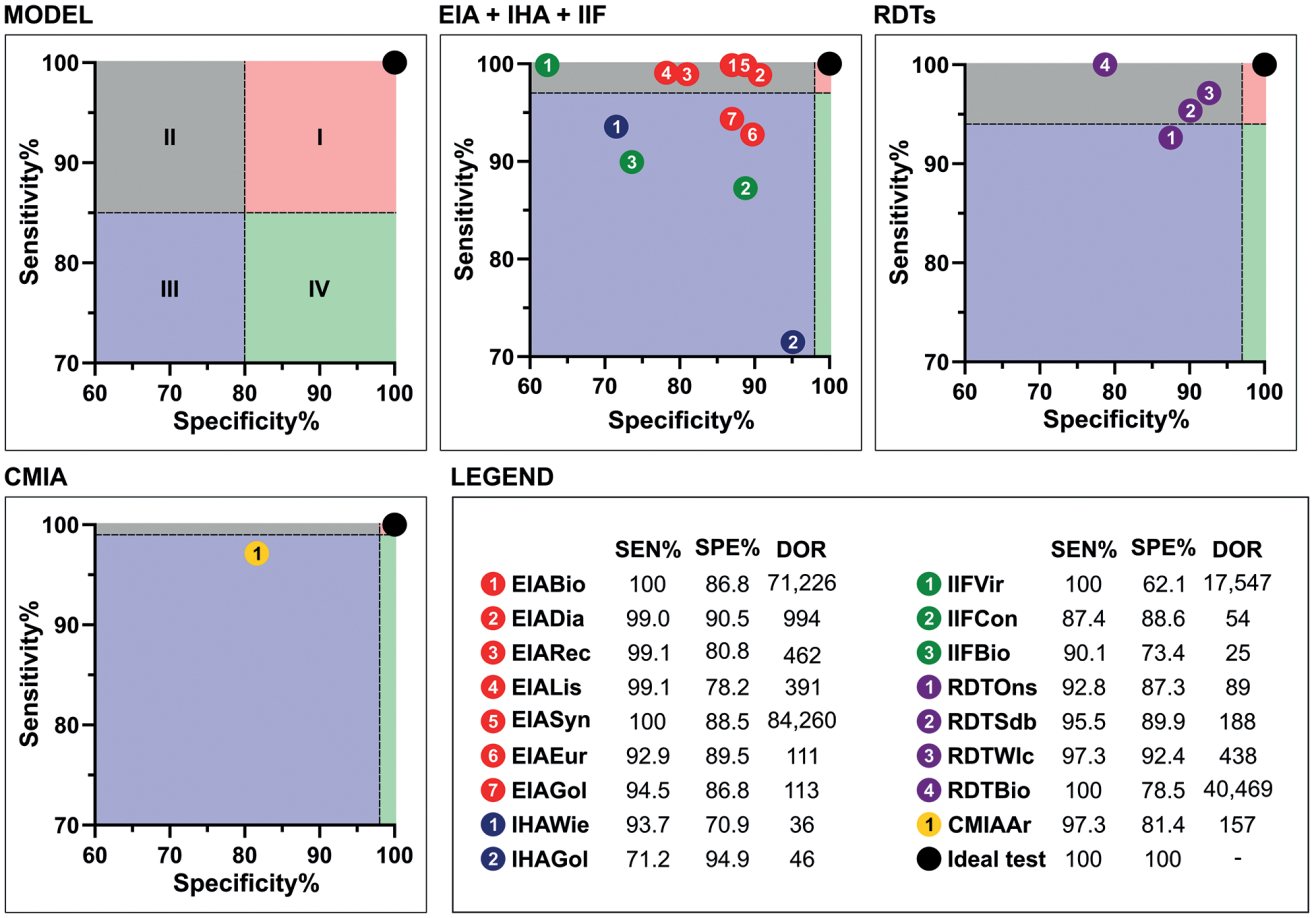
sensitivity vs. specificity data for the 18 kits licensed for use and commercially available in Brazil, as grouped in this study. The black dot serves as a reference point for an ideal diagnostic test with 100% accuracy.

In Fig. 1, the relationship between specificity and sensitivity for all 17 evaluated kits is illustrated. The figure includes an ideal test point (represented as a black dot at 100% accuracy) for comparison and is divided into four quadrants based on a graphical model. None of the evaluated tests satisfied the criteria to be placed in quadrant I, as defined by the PAHO guidelines.^4^

Among the EIA kits, EIADia and EIASym demonstrated the closest approximation to an ideal test. Similarly, RDTWlc and RDTSdb exhibited the best performance among the rapid diagnostic tests. In contrast, all IHA and IIF tests were notably distant from quadrant I. The CMIA assay showed high sensitivity; however, this was accompanied by lower specificity, limiting its classification as an ideal diagnostic test.

*Analysis of diagnostic kit combinations* - To address diagnostic uncertainty and enhance both sensitivity and specificity, pairwise analyses were conducted using individual test data from Table I. Since the recommended diagnostic procedure involves combining tests,^3^ we analysed three scenarios that used combinations of two different diagnostic platforms. These results are summarised in Table III for combinations of EIA with other laboratory-based tests (IHA, IIF, or CMIA), IHA with IIF or CMIA, and IIF with CMIA. Additionally, Table IV explores the combination of RDTs with other diagnostic tests.

**TABLE III:** Performance parameters for the pairwise analysis using serological laboratorial-based commercial methods in Brazil for detecting anti-*Trypanosoma cruzi* antibodies

Pairwise analysis	SEN % (IC95%)	SPE % (IC95%)	ACC % (IC95%)
Enzyme immunoassay			
EIABio + IHAWie	100 (99.6-100)	61.5 (46.5-73.9)	88.1 (83.1-91.9)
EIABio + IHAGol	100 (98.7-100)	82.4 (67.9-90.8)	90.7 (82.4-95.1)
EIABio + IIFVir	100 (99.9-100)	53.9 (38.8-67.5)	85.7 (80.9-89.9)
EIABio + IIFCon	100 (99.3-100)	76.9 (61.7-87.0)	90.1 (83.1-94.4)
EIABio + IIFBio	100 (99.4-100)	63.7 (48.6-75.9)	87.3 (81.6-91.6)
EIABio + CMIAAr	100 (99.7-100)	70.7 (53.9-82.8)	91.2 (86.0-94.8)
EIADia + IHAWie	99.9 (99.4-100)	64.2 (49.1-76.0)	88.8 (83.8-92.5)
EIADia + IHAGol	99.7 (98.0-100)	85.9 (71.7-93.4)	92.4 (84.0-96.5)
EIADia + IIFVir	100 (99.8-100)	56.2 (40.9-69.4)	86.4 (81.6-90.5)
EIADia + IIFCon	99.9 (99.0-100)	80.2 (65.1-89.5)	91.4 (84.4-95.5)
EIADia + IIFBio	99.9 (99.1-100)	66.4 (51.3-78.1)	88.2 (82.4-92.3)
EIADia + CMIAAr	100 (99.6-100)	73.7 (56.9-85.1)	92.1 (86.8-95.5)
EIARec + IHAWie	99.9 (99.4-100)	57.3 (42.5-70.1)	86.7 (81.8-90.7)
EIARec + IHAGol	99.7 (98.1-100)	76.7 (62.0-86.2)	87.5 (79.0-92.7)
EIARec + IIFVir	100 (99.8-100)	50.2 (35.4-64.1)	84.6 (79.9-88.9)
EIARec + IIFCon	99.9 (99.0-100)	71.6 (56.3-82.6)	87.7 (80.7-92.5)
EIARec + IIFBio	99.9 (99.2-100)	59.3 (44.4-72.1)	85.7 (80.0-90.2)
EIARec + CMIAAr	100 (99.6-100)	65.8 (49.2-78.6)	89.7 (84.5-93.6)
EIALis + IHAWie	99.9 (99.4-100)	55.4 (40.7-68.5)	86.1 (81.2-90.2)
EIALis + IHAGol	99.7 (98.1-100)	74.2 (59.5-84.2)	86.2 (77.6-91.6)
EIALis + IIFVir	100 (99.8-100)	48.6 (34.0-62.5)	84.1 (79.4-88.4)
EIALis + IIFCon	99.9 (99.0-100)	69.3 (54.0-80.7)	86.7 (79.7-91.7)
EIALis + IIFBio	99.9 (99.2-100)	57.4 (42.6-70.4)	85.0 (79.4-89.6)
EIALis + CMIAAr	100 (99.6-100)	63.7 (47.2-76.7)	89.1 (83.9-93.0)
EIASym + IHAWie	100 (99.6-100)	62.7 (47.8-74.8)	88.5 (83.5-92.2)
EIASym + IHAGol	100 (98.7-100)	84.0 (69.7-91.9)	91.5 (83.3-95.7)
EIASym + IIFVir	100 (99.9-100)	55.0 (39.8-68.3)	86.0 (81.3-90.2)
EIASym + IIFCon	100 (99.3-100)	78.4 (63.4-88.1)	90.7 (83.9-94.9)
EIASym + IIFBio	100 (99.4-100)	65.0 (49.9-76.8)	87.7 (82.1-91.9)
EIASym + CMIAAr	100 (99.7-100)	72.0 (55.3-83.8)	91.6 (86.4-95.1)
EIAEur + IHAWie	99.6 (98.3-99.0)	63.5 (48.4-75.4)	88.4 (82.8-92.3)
EIAEur + IHAGol	98.0 (94.7-99.3)	84.9 (70.7-92.7)	91.1 (82.0-95.8)
EIAEur + IIFVir	100 (99.5-100)	55.6 (40.4-68.9)	86.2 (81.2-90.3)
EIAEur + IIFCon	99.1 (97.2-99.7)	79.3 (64.2-88.8)	90.6 (83.0-95.0)
EIAEur + IIFBio	99.3 (97.6-99.8)	65.7 (50.6-77.5)	87.5 (81.2-92.0)
EIAEur + CMIAAr	99.8 (98.9-100)	72.9 (56.1-84.5)	91.7 (86.1-95.3)
EIAGol +IHAWie	99.7 (98.6-99.9)	61.5 (46.5-73.9)	87.8 (82.4-91.8)
EIAGol + IHAGol	98.4 (95.6-99.5)	82.4 (67.9-90.8)	89.9 (80.9-94.9)
EIAGol + IIFVir	100 (99.6-100)	53.9 (38.8-67.5)	85.7 (80.7-89.9)
EIAGol + IIFCon	99.3 (97.7-99.8)	76.9 (61.7-87.0)	89.7 (82.2-94.3)
EIAGol + IIFBio	99.5 (98.1-99.9)	63.7 (48.6-75.9)	86.9 (80.7-91.5)
EIAGol + CMIAAr	99.9 (99.1-100)	70.7 (53.9-82.8)	91.1 (85.5-94.8)
Indirect haemagglutination			
IHAWie + IIFVir	100 (99.6-100)	44.0 (30.1-58.0)	82.6 (78.0-87.0)
IHAWie + IIFCon	99.2 (97.5-99.8)	62.8 (47.9-74.8)	87.9 (82.1-92.0)
IHAWie + IIFBio	99.4 (97.9-99.8)	52.0 (37.7-65.3)	84.7 (79.3-89.1)
IHAWie + CMIAAr	99.8 (99.1-100)	57.7 (41.8-71.2)	86.8 (81.3-91.0)
IHAGol + IIFVir	100 (98.7-100)	58.9 (43.9-71.3)	78.2 (69.7-84.8)
IHAGol + IIFCon	96.4 (92.4-98.4)	84.1 (69.9-92.0)	89.9 (80.5-95.0)
IHAGol + IIFBio	97.1 (93.6-98.8)	69.7 (55.1-80.3)	82.6 (73.2-89.0)
IHAGol + CMIAAr	99.2 (97.1-99.8)	77.2 (61.0-87.5)	87.6 (78.0-93.3)
Indirect immunofluorescence			
IIFVir + CMIAAr	100 (99.7-100)	50.5 (34.9-65.0)	84.7 (79.6-89.2)
IIFCon + CMIAAr	99.7 (98.5-99.9)	72.1 (55.5-83.9)	87.8 (80.0-93.0)
IIFBio + CMIAAr	99.7 (98.7-99.9)	59.7 (43.7-73.1)	85.7 (79.5-90.6)

SEN: sensitivity; SPE: specificity; ACC: accuracy.

**TABLE IV:** Performance parameters for the pairwise analysis using rapid diagnostic tests and serological laboratorial-based commercial methods in Brazil for detecting anti-*Trypanosoma cruzi* antibodies

Pairwise analysis	SEN % (IC95%)	SPE % (IC95%)	ACC % (IC95%)
RDTOns + EIABio	100 (99.5-100)	75.8 (60.4-87.0)	92.0 (86.6-95.7)
RDTOns + EIADia	99.9 (99.3-100)	79.0 (63.7-89.4)	92.4 (86.5-96.2)
RDTOns + EIARec	99.9 (99.3-100)	70.5 (55.1-82.5)	90.2 (84.7-94.2)
RDTOns + EIALis	99.9 (99.3-100)	68.3 (52.9-80.6)	89.2 (83.5-93.4)
RDTOns + EIASym	100 (99.5-100)	77.3 (62.0-88.0)	92.7 (87.5-96.2)
RDTOns + EIAEur	99.5 (98.1-99.9)	78.1 (62.9-88.7)	90.1 (82.6-95.0)
RDTOns + EIAGol	99.6 (98.4-99.9)	75.8 (60.4-87.0)	90.3 (83.6-94.9)
RDTOns + IHAWie	99.5 (98.3-99.9)	61.9 (46.9-74.8)	87.9 (82.4-92.1)
RDTOns + IHAGol	97.9 (94.8-99.3)	82.8 (68.4-91.9)	89.9 (80.8-95.4)
RDTOns + IIFVir	100 (99.5-100)	54.2 (39.1-68.3)	85.8 (80.8-90.2)
RDTOns + IIFCon	99.1 (97.2-99.8)	77.3 (62.2-88.1)	89.7 (82.2-94.7)
RDTOns + IIFBio	99.3 (97.7-99.8)	64.1 (49.0-76.8)	87.0 (80.6-91.8)
RDTOns + CMIAAr	99.8 (99.0-100)	71.1 (54.3-83.8)	91.2 (85.6-95.1)
RDTSdb + EIABio	100 (99.7-100)	78.0 (62.7-88.5)	92.8 (87.5-96.2)
RDTSdb + EIADia	100 (99.5-100)	81.4 (66.2-91.0)	93.3 (87.5-96.8)
RDTSdb + EIARec	100 (99.5-100)	72.6 (57.3-84.0)	90.9 (85.6-94.7)
RDTSdb + EIALis	100 (99.5-100)	70.3 (54.9-82.0)	89.9 (84.3-93.9)
RDTSdb + EIASym	100 (99.7-100)	79.6 (64.4-89.6)	93.5 (88.4-96.7)
RDTSdb + EIAEur	99.7 (98.6-99.9)	80.5 (65.3-90.3)	91.2 (83.9-95.7)
RDTSdb + EIAGol	99.8 (98.8-100)	78.0 (62.7-88.5)	91.3 (84.7-95.5)
RDTSdb + IHAWie	99.7 (98.7-100)	63.7 (48.7-76.1)	88.6 (83.2-92.6)
RDTSdb + IHAGol	98.7 (96.2-99.7)	85.3 (71.0-93.6)	91.6 (82.9-96.5)
RDTSdb + IIFVir	100 (99.6-100)	55.8 (40.6-69.5)	86.3 (81.3-90.6)
RDTSdb + IIFCon	99.4 (98.0-99.9)	79.7 (64.6-89.7)	90.9 (83.6-95.5)
RDTSdb + IIFBio	99.6 (98.3-99.9)	66.0 (50.9-78.2)	87.8 (81.7-92.3)
RDTSdb + CMIAAr	99.9 (99.2-100)	73.2 (56.4-85.3)	91.9 (86.4-95.6)
RDTWlc + EIABio	100 (99.7-100)	80.2 (65.2-90.1)	93.5 (88.3-96.7)
RDTWlc + EIADia	100 (99.6-100)	83.6 (68.8-92.6)	94.1 (88.5-97.3)
RDTWlc + EIARec	100 (99.6-100)	74.7 (59.5-85.5)	91.6 (86.4-95.2)
RDTWlc + EIALis	100 (99.6-100)	72.3 (57.1-83.5)	90.6 (85.2-94.4)
RDTWlc + EIASym	100 (99.7-100)	81.8 (66.9-91.2)	94.2 (89.2-97.2)
RDTWlc + EIAEur	99.8 (98.9-100)	82.7 (67.9-92.0)	92.3 (85.3-96.4)
RDTWlc + EIAGol	99.9 (99.1-100)	80.2 (65.2-90.1)	92.2 (85.9-96.1)
RDTWlc + IHAWie	99.8 (99.0-100)	65.5 (50.6-77.5)	89.2 (84.0-93.0)
RDTWlc + IHAGol	99.2 (97.1-99.9)	87.7 (73.8-95.3)	93.1 (84.8-97.4)
RDTWlc + IIFVir	100 (99.7-100)	57.4 (42.2-70.8)	86.8 (81.9-90.9)
RDTWlc + IIFCon	99.7 (98.5-100)	81.9 (67.1-91.3)	92.0 (85.0-96.2)
RDTWlc + IIFBio	99.7 (98.7-100)	67.8 (52.9-79.6)	88.6 (82.7-92.8)
RDTWlc + CMIAAr	99.9 (99.4-100)	75.2 (58.6-86.8)	92.5 (87.2-96.0)
RDTBio + EIABio	100 (99.9-100)	68.1 (52.8-79.8)	89.5 (84.3-93.3)
RDTBio + EIADia	100 (99.8-100)	71.0 (55.7-82.1)	89.6 (83.9-93.5)
RDTBio + EIARec	100 (99.8-100)	63.4 (48.2-75.8)	87.9 (82.8-92.0)
RDTBio + EIALis	100 (99.8-100)	61.4 (46.2-74.0)	86.9 (81.6-91.1)
RDTBio + EIASym	100 (99.9-100)	69.5 (54.2-80.8)	90.2 (85.3-93.8)
RDTBio + EIAEur	100 (99.5-100)	70.3 (55.0-81.5)	86.9 (79.9-91.8)
RDTBio + EIAGol	100 (99.6-100)	68.1 (52.8-79.8)	87.6 (81.4-92.1)
RDTBio + IHAWie	100 (99.6-100)	55.7 (41.0-68.6)	86.3 (81.4-90.3)
RDTBio + IHAGol	100 (98.7-100)	74.5 (59.8-84.4)	86.5 (78.1-91.7)
RDTBio + IIFVir	100 (99.9-100)	48.7 (34.2-62.7)	84.1 (79.5-88.4)
RDTBio + IIFCon	100 (99.3-100)	69.6 (54.4-80.8)	86.9 (80.0-91.8)
RDTBio + IIFBio	100 (99.4-100)	57.6 (42.8-70.5)	85.2 (79.6-89.7)
RDTBio + CMIAAr	100 (99.7-100)	63.9 (47.5-76.9)	89.2 (84.1-93.1)

SEN: sensitivity; SPE: specificity; ACC: accuracy.

Our findings reveal that sensitivity consistently increased when test results were evaluated in parallel, compared to individual tests such as EIAEur, EIAGol, IHAWie, IHAGol, IIFCon, IIFBio, RDTOns, and RDTSdb. This aligns with the expected outcome of parallel testing, which detects more true positives but also introduces more false positives. In contrast, specificity values were generally lower.

All EIA-based combinations achieved sensitivity values exceeding 99% (Table III), except for the EIAEur + IHAGol pair (98%) and EIAGol + IHAGol (98.4%). Maximum sensitivity was observed in several combinations, particularly those using EIABio or EIASym, regardless of the second methodology. Despite these minor variations, no significant differences in sensitivity were found among the pairs. Conversely, specificity values varied widely, ranging from 48.6% for EIALis + IIFVir to 85.9% for EIADia + IHAGol. Due to these variations, accuracy values exceeded 90% for only a few combinations, such as EIABio, EIADia, or EIASym paired with IHAGol, IIFCon, or CMIAAr, and EIAGol combined with CMIAAr (91.1%). Interestingly, no combination involving EIARec, EIALis, or EIAGol (except EIAGol + CMIAAr) surpassing 90% of accuracy.

Table III also presents the diagnostic performance of IHA combined with IIF or CMIA tests. Sensitivity values ranged from 96.4% (IHAGol + IIFCon) to 100% (IHAWie + IIFVir or IHAGol + IIFVir). A significant difference in sensitivity was noted only for IHAGol + IIFCon compared to combinations achieving 100% sensitivity. Specificity values also varied widely, from 44% (IHAWie + IIFVir) to 84.1% (IHAGol + IIFCon). Combinations involving IHAWie did not exceed 65% specificity. Accuracy values for IHA pairs ranged from 82.6% to 89.9%, except for IHAGol + IIFVir, which had an accuracy of 78.2%.

The analysis of IIF combined with CMIA showed sensitivity values exceeding 99.7% (Table III), while specificity values ranged from 50.5% (IIFVir + CMIAAr) to 72.1% (IIFBio + CMIAAr). Accuracy values for IIF combinations were relatively consistent, averaging approximately 85%.

The pairwise analysis involving the combination of RDTs with laboratory-based methodologies is presented in Table IV. Elevated sensitivity values were observed for all combinations with EIA, IIF, IHA, and CMIA kits. The lowest sensitivity values were noted for RDTOns + IHAGol (97.9%) and RDTSdb + IHAGol (98.7%), with significant differences only when compared to combinations achieving 100% sensitivity. Notably, RDTBio consistently reached 100% sensitivity regardless of the diagnostic platform used in combination.

Specificity values for the RDT combinations ranged from 48.7% (RDTBio + IIFVir) to 87.7% (RDTWlc + IHAGol). Among the RDTs, RDTBio exhibited the lowest specificity and accuracy values; however, these were not significantly different from other RDTs. Overall, lower accuracy was observed when any RDT was combined with IHAWie, IIFVir or IIFBio, highlighting potential limitations of these combinations in maintaining balanced diagnostic performance.

**Fig. 2:**
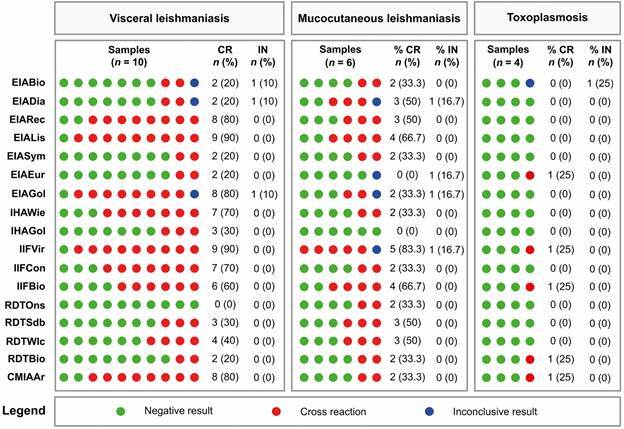
analysis of cross-reactivity using 18 commercial tests for Chagas disease (CD) diagnosis in sera from individuals with visceral leishmaniasis (left panel), mucocutaneous leishmaniasis (middle panel), and toxoplasmosis (right panel). CR: cross-reactivity; IN: undetermined; N: number of samples.

*Cross reactivity analysis* - We assessed antigenic cross-reactivity among antibodies from various parasitic diseases using all 17 commercial kits. The analysis included a panel of 20 serum samples, and individual data points are detailed in Supplementary data (Table). Results are summarised in Fig. 2.

For serum samples from individuals with visceral leishmaniasis (n = 10), no cross-reactivity or inconclusive results were observed when using RDTOns exclusively. However, the EIABio, EIADia, and EIAGol kits identified one sample (10%) within the grey zone. Cross-reactivity rates varied significantly among the tests, ranging from 20% (EIABio, EIADia, EIASym, EIAEur, and RDTBio) to 90% (EIALis). Intermediate rates included 30% (IHAGol and RDTSdb), 40% (RDTWlc), 60% (IIFBio), 70% (IHAWie and IIFCon), and 80% (EIARec, EIAGol, and CMIAAr). Kits utilising crude antigens exhibited notably higher cross-reactivity rates.

For samples from individuals with mucocutaneous leishmaniasis (n = 10), one sample (16.7%) fell within the grey zone when tested with EIADia, EIAEur, EIAGol, and IIFVir. Cross-reactivity was observed in two samples (33.3%) when tested with EIABio, EIASym, EIAGol, IHAWie, IIFCon, RDTOns, RDTBio, and CMIAAr. Three samples exhibited cross-reactivity with EIADia, EIARec, RDTSdb, and RDTWlc, while four samples cross-reacted with EIALis and IIFBio. Remarkably, five samples showed cross-reactivity with IIFVir, and none of the samples were accurately classified by this kit. In contrast, IHAGol demonstrated no cross-reactivity or inconclusive results.

For toxoplasmosis-positive samples, no positive or inconclusive results were observed when tested with EIADia, EIARec, EIALis, EIASym, EIAGol, IHAWie, IHAGol, IIFCon, RDTOns, RDTSdb, or RDTWlc. EIABio produced an inconclusive result for one sample (25%), while cross-reactivity was observed with EIAEur, IIFVir, IIFBio, and CMIAAr for the same sample.

**Fig. 3:**
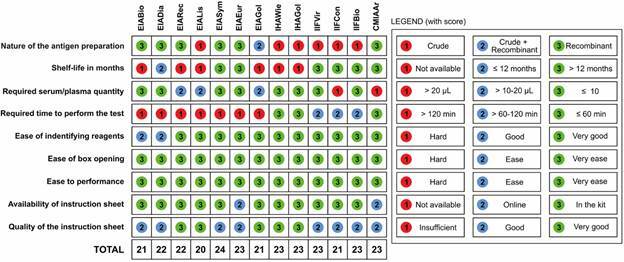
usability assessment enzyme immunoassays (EIA), indirect immunofluorescence assays (IIF), indirect haemagglutination (IHA), and chemiluminescence microparticle immunoassays (CMIA) kits investigated in this study.

*Usability assessment* - The usability assessment evaluated the ease of performing serological tests and interpreting results for each kit based on various criteria (Fig. 3). Among the kits, EIASym achieved the highest usability score of 24 points, narrowly missing the maximum score due to the significant time required for the assay and the medium quality of its instruction sheet. Following EIASym, six kits scored 23 points (EIAEur, IHAWie, IHAGol, IIFVir, IIFBio, and CMIAAr), two kits scored 22 points (EIADia and EIARec), three kits scored 21 points (EIABio, EIAGol, and IIFCon), and one kit scored 20 points (EIALis).

Six kits (EIALis, IHAWie, IHAGol, IIFVir, IIFCon, and IIFBio) used crude antigen preparations, while another six kits (EIABio, EIADia, EIARec, EIASym, EIAEur, and CMIAAr) employed recombinant antigens. Only one kit (EIAGol) utilised a combination of crude and recombinant antigens for the detection of anti-*T. cruzi* antibodies. Importantly, information on shelf life was unavailable for six kits (EIABio, EIARec, EIALis, EIAGol, IHAWie, and IHAGol).

Sample volume requirements varied significantly among the kits. High sample volumes exceeding 20 µL were required by IIFBio and CMIAAr, while EIARec, EIALis, and EIAGol required moderate volumes between 10 and 20 µL. The remaining tests required low sample volumes of 10 µL or less. Regarding test duration, only the IHAWie, IHAGol, and CMIAAr provided results within 1 h. All tests performed well in terms of ease of identifying reagents, opening the kit box, ease of assay performance, and quality of the instruction sheet. However, the instruction sheets for EIAEur and CMIAAr kits were only available online, whereas all other kits included printed instructions within the packaging.

## DISCUSSION

This study provides a comprehensive evaluation of 17 serological tests for detecting anti-*T. cruzi* antibodies, encompassing diverse platforms including EIA, IHA, IIF, RDT, and CMIA. The findings reveal significant variability in sensitivity, specificity, and accuracy across these kits, with critical implications for clinical practice and public health strategies in managing CD.

Substantial differences in sensitivity and specificity were observed across diagnostic platforms. Kits such as EIABio, EIASym, IIFVir, and RDTBio demonstrated 100% sensitivity, making them highly effective for screening in endemic areas where identifying all cases is essential. These findings align with previous studies reporting EIA sensitivity ranges of 97.3-100%.^12^ However, specificity was a major concern for tests like IHAWie and IIFVir, which exhibited lower specificity and a higher likelihood of false positives. This issue is particularly problematic in low-prevalence settings, where false positives can lead to unnecessary follow-up testing, increased costs, and psychological distress for individuals. Furthermore, the lower-than-expected positive likelihood ratios for many tests indicate that a positive result does not always reliably confirm disease presence. Achieving a balance between sensitivity and specificity is therefore critical, especially in resource-limited settings. While maximising sensitivity is essential for effective screening, improving specificity is equally important to enhance diagnostic accuracy and reduce the consequences of false positives.

Our findings are consistent with a U.S. study evaluating the performance of three FDA-cleared EIAs and one rapid test (InBios) for CD.^13^ The InBios rapid test exhibited high sensitivity (97.4-99.3%), confirming its utility as a screening tool. Conversely, the Hemagen EIA had lower sensitivity (88.0-92.0%), while Ortho and Wiener EIAs demonstrated intermediate sensitivity (92.4-96.5% and 94.0-97.1%, respectively). Specificity varied throughout these tests, highlighting the trade-off between identifying all true positives and minimising false positives. Although there are no universal guidelines for defining high or low sensitivity and specificity, the PAHO reported EIA sensitivity of 97% (95% CI: 96-98) and specificity of 98% (95% CI: 97-99).^4^ Similar data for IHA and IIF tests are lacking, emphasising the need for careful test selection based on epidemiological context and testing purpose (*e.g.*, screening, confirmation, or surveillance).

Among diagnostic platforms, EIA consistently exhibited the highest sensitivity, specificity, and DORs in this study. These tests are capable of detecting low antibody titters, are easy to perform, provide objective results, and allow for high-throughput sample processing. However, they require specialised laboratory infrastructure. Kits using crude or recombinant antigens showed comparable performance, although differences in antigenic composition and reagent quality can influence results. Cross-reactivity with other diseases remains a challenge and highlights the need for more specific antigens to capture *T. cruzi* antibodies accurately.

Indirect haemagglutination assays are easy to perform, allow for the simultaneous testing of many samples, and do not require specialised equipment, but their accuracy is compromised by the subjective interpretation of results. The two evaluated IHA tests had similar components and characteristics, despite their different performances. Among the two IHA kits evaluated, IHAWie showed higher sensitivity (93.7%) but the lowest specificity (70.9%), while IHAGol exhibited high specificity (94.9%) but lower sensitivity (71.2%). The lower number of cross-reactions with the IHAGol can be explained by its high specificity. According to the sensitivity and specificity values reported by PAHO for EIA,^4^ adopted in this study for IHA and IIF tests, none of the IHA kits reached the minimum reported sensitivity and specificity. This discrepancy underscores the importance of antigenic composition and test design in minimising cross-reactions and false positives.

Indirect immunofluorescence assays can detect low antibody titters but require a well-equipped laboratory, expensive equipment that demands periodic maintenance, and skilled personnel. Additionally, their results are subjectively interpreted, which can impact accuracy. The evaluated IIF kits demonstrated variability in their components and characteristics, despite all using *T. cruzi* suspensions as the capture antigen. However, critical details such as the preparation method or the strain used were not provided. The IIFVir kit achieved a sensitivity of 100%, but its specificity was the lowest among all evaluated tests, at 62.1%. This low specificity is likely due to cross-reactions with other diseases, potentially stemming from the use of crude antigens. When compared to the sensitivity and specificity thresholds reported by PAHO for EIA, the IIF tests exceeded the sensitivity values but failed to meet the minimum specificity standards.

The CMIA, characterised by full automation and objective interpretation, demonstrated high sensitivity (97.3%) but moderate specificity (81.4%) in this study. This profile is particularly suitable for haemotherapy services, where identifying all positive cases is critical. PAHO and the WHO recommend using highly sensitive assays (above 99%), such as EIA and CMIA, for CD screening in such settings.^4^ ^,^ ^14^

Erroneous diagnoses in chronic CD often stem from factors such as the genetic diversity of *T. cruzi*,^15^ the choice of antigens used in tests,^12^ variability in disease prevalence,^16^ ^,^ ^17^ and individual immune responses to infection.^18^ In response to these challenges, international guidelines recommend employing two serological tests with distinct antigenic preparations to enhance sensitivity and reduce the number of undiagnosed cases.^4^ ^,^ ^19^ Using two tests in parallel generally increases sensitivity and ensures fewer missed diagnoses. While most serological tests used for this purpose exhibit high sensitivity and specificity, particularly in immunocompetent individuals, discrepancies between tests may still occur due to differences in accuracy and execution.

To address the limitations of individual tests, this study conducted pairwise analyses combining different diagnostic platforms. Results showed that pairing EIA with other platforms such as IHA, IIF, or CMIA generally improved sensitivity, often exceeding 99%. However, specificity varied significantly, influencing overall accuracy. For instance, combinations like EIADia + IHAGol and EIADia + IIFCon achieved higher accuracy, whereas combinations involving IHAWie or IIFVir yielded lower specificity and accuracy. When incorporating RDTs into the combinations, RDTBio consistently demonstrated 100% sensitivity, regardless of the paired test. However, the highest specificity values were observed when RDTWlc or RDTSdb were combined with IHAGol, achieving 87.7% and 85.3% specificity, respectively. Notably, accuracy values exceeding 94% were obtained when RDTWlc was paired with EIASym or EIADia.

The use of RDTs as the initial step in diagnostic algorithms is crucial for expanding access to diagnosis, particularly in remote or underserved areas. However, it is important to recognise that parallel testing, while enhancing sensitivity, often compromises specificity. To mitigate these challenges, the Ministry of Health has implemented a diagnostic strategy requiring two combined tests to confirm outcomes. According to this protocol, both tests must yield positive concordant results, either positive or negative, for a definitive diagnosis. If results are discordant, a third test employing different methodology is required to establish the outcome.^3^ Considering these trade-offs, selecting optimal test combinations is critical, particularly in resource-constrained settings where cost-effectiveness is a primary concern. Further research is needed to identify the most effective and sustainable diagnostic strategies for such environments.

Cross-reactivity remains a significant challenge, as evidenced by our analysis. The observed cross-reactivity with sera from individuals with other parasitic diseases illustrates the complex antigenic landscape of *T. cruzi* and related pathogens. This issue is particularly critical in regions with high prevalence of co-infections, where accurate differential diagnosis is essential to avoid misdiagnosis and ensure appropriate treatment. Our findings underscore the need for the development of more specific antigens and the refinement of testing platforms to improve specificity without compromising sensitivity.

When employing diagnostic kits in epidemiological surveys, it is essential to consider not only specificity and sensitivity but also positive predictive values (PPVs), which are heavily influenced by the disease prevalence in the tested population. In this study, most tests demonstrated higher sensitivity than specificity, particularly in parallel testing strategies where sensitivity is prioritised. Consequently, the negative likelihood ratios of many tests were more favourable, suggesting that a negative result has a high probability of being accurate. This implies that, in cases of low clinical suspicion or in populations with low disease prevalence, where the negative predictive value is relatively high, a negative test result may suffice without the need for additional confirmatory testing. However, in populations with higher disease prevalence or elevated clinical suspicion, confirmatory testing may still be necessary to rule out false negatives. For confirmation of negative tests, using tests with lower specificity than the screening test should be avoided to reduce the risk of false-positive results, while for positive tests, lower-sensitivity tests may introduce false negatives if not carefully selected.

To establish an optimal approach for the use of commercially available diagnostic kits for chronic CD, conducting a cost-effectiveness analysis (CEA) is essential. CEA plays a critical role in diagnostics, as the wide variability in diagnostic alternatives and their substantial resource requirements demand informed decision-making. In this context, CEAs are invaluable for prioritising strategies and have been recommended by the PAHO to support the implementation of rapid diagnostic tests across the Americas. By defining priority epidemiological scenarios, CEAs can provide crucial insights to guide the selection of diagnostic strategies, helping decision-makers determine which tests to employ and in which geographic areas they should be implemented.

This study provides a comprehensive evaluation of the performance of 17 serological diagnostic kits for CD, uncovering significant variability in their sensitivity, specificity, and cross-reactivity. While some tests demonstrated excellent sensitivity, challenges related to specificity and cross-reactivity highlight the limitations of many available kits, particularly those using crude antigens. These findings emphasise the importance of selecting diagnostic tools tailored to specific epidemiological contexts and the critical need for combining tests in parallel to enhance diagnostic accuracy. While this study provides insights into diagnostic accuracy within the sampled population, further validation in diverse geographic settings is necessary to confirm the applicability of these findings to broader populations. Future studies incorporating samples from multiple endemic areas with distinct epidemiological profiles would strengthen the generalisability of the results and ensure that the evaluated diagnostic methods perform reliably across different regions. Additionally, future research should focus on the development of novel diagnostic antigens, the optimisation of test combinations, and the rigorous evaluation of these strategies in field settings. Such advancements are essential for improving the accuracy and accessibility of CD diagnostics, with far-reaching implications for enhancing patient care and advancing public health initiatives to combat this neglected tropical disease.
